# Ewing sarcoma of the liver with multilocular cystic mass formation: a case report

**DOI:** 10.1186/s12885-015-1017-3

**Published:** 2015-01-22

**Authors:** Yukinori Ozaki, Yuji Miura, Shigehiro Koganemaru, Koichi Suyama, Naoko Inoshita, Takeshi Fujii, Masaji Hashimoto, Tetsuo Tamura, Kazuo Takeuchi, Toshimi Takano

**Affiliations:** 1 Department of Medical Oncology, Toranomon Hospital, 2-2-2 Toranomon, Minato-ku, Tokyo 105-8470 Japan; 2 Department of Pathology, Toranomon Hospital, Tokyo, Japan; 3 Department of Digestive Surgery, Toranomon Hospital, Tokyo, Japan; 4 Department of Gastrointestinal Medicine, Toranomon Hospital, Tokyo, Japan

**Keywords:** Ewing sarcoma, Primitive neuroectodermal tumor, Cysts, Liver Neoplasms, Chemotherapy

## Abstract

**Background:**

Ewing sarcoma is a rare tumor that occurs commonly in the long bones of children or adolescents that can also arise in soft tissues including the extremities, retroperitoneum, chest wall, and rarely in the liver as primary sites. We report a case of Ewing sarcoma arising primarily in the liver and, to our knowledge, this is the fourth reported case of Ewing sarcoma occurring in the liver.

**Case presentation:**

A 27-year-old Japanese woman was admitted with sudden onset right upper abdominal pain. Clinical examination revealed a multilocular cystic mass consisting of thickened, irregular septa and nodal walls in the right hepatic lobe. Ultrasound-guided aspiration biopsy of the liver mass showed clusters of small atypical round cells and the clinical preoperative diagnosis was mucinous cystadenoma of the liver. The patient underwent an extended right hepatectomy and histopathological findings revealed sheet-like proliferation of small- to medium-sized round cells. Tumor cells were positive for periodic acid-Schiff reaction and immunoreactive for glycoprotein C99 and gene NKX2.2, as well as the neuroendocrine markers, CD56 and synaptophysin. EWS-FLI-1 fusion transcript type 1 was detected by reverse transcriptase polymerase chain reaction. Pathological and molecular analysis confirmed the diagnosis of Ewing sarcoma arising primarily in the liver and the patient received adjuvant systemic chemotherapy with vincristine, doxorubicin, and cyclophosphamide, alternating with ifosfamide and etoposide. We found no evidence of recurrence 15 months after completing chemotherapy.

**Conclusion:**

We present an extremely rare case of Ewing sarcoma arising primarily in the liver. To our knowledge, this is the fourth reported case of Ewing sarcoma occurring in the liver, and the first case with a multilocular cystic liver mass. Imaging examinations of the other three reported cases showed solid tumors and a diffuse enlarged liver without mass lesion. Clinicians should consider the possibility of Ewing sarcoma in young patients with a multilocular cystic mass with thick and/or irregular cyst walls in the liver.

## Background

The Ewing sarcoma family of tumors (EFTs) is a rare entity that includes extraosseous Ewing sarcoma (ES), primitive neuroectodermal tumor (PNET), Askin tumor, and atypical ES. ES represents the second most common primary bone malignancy of children and young adults [1] and most often arises in the long bones of the extremities and the pelvic bones [2]. ES rarely occurs in soft tissues without bone involvement. Common primary sites of extraosseous ES are the deep soft tissues of the extremities, retroperitoneum, and chest wall. Extraosseous ES has also been reported to arise in the kidney [3], uterus [4], gastrointestinal tract [5], and other visceral organs. To our knowledge, only three published case reports discuss ES arising in the liver [6–8]. We report a case of a 27-year-old female with ES arising in the liver and we review the literature on extraosseous ES [9].

## Case presentation

### Case report

**Figure 1:**
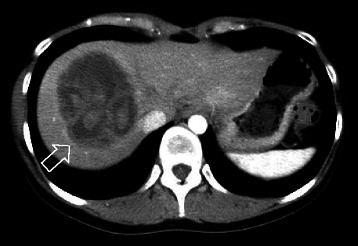
**Enhanced computed tomography scan of the abdomen showing the multilocular cystic mass.** Enhanced computed tomography scan showing a multilocular cystic mass (arrow) with enhanced septa, measuring 82 × 66 mm in liver segment 8.

A 27-year-old previously healthy woman was transferred to the emergency department of another hospital in June 2012 because of sudden onset right upper abdominal pain radiating to her back. She was afebrile and other vital signs were normal. Physical examination revealed no abnormality except for right upper abdominal tenderness. Enhanced computed tomography (CT) scan revealed a multilocular cystic mass with enhanced septa, measuring 82 × 66 mm in liver segment 8 (Figure 1). No other lesion was detected in the CT scan. Ultrasound-guided aspiration biopsy of the liver mass obtained bloody, mucinous fluid and bacterial examination of the fluid revealed that it was sterile. Cytological examination showed clusters of small round atypical cells with high nuclear/cytoplasm ratio, but the diagnosis was inconclusive. Blood examination showed leukocytosis (white blood cells, 13.0 × 10^3^/μL) with normal transaminase levels and negative hepatitis B surface antigen and antibody. Serum antibodies for hepatitis C virus, human immunodeficiency virus, and Entamoeba histolytica were negative. Levels of tumor markers of carcinoembryonic antigen and carbohydrate antigen 19–9 were within normal limits.

**Figure 2:**
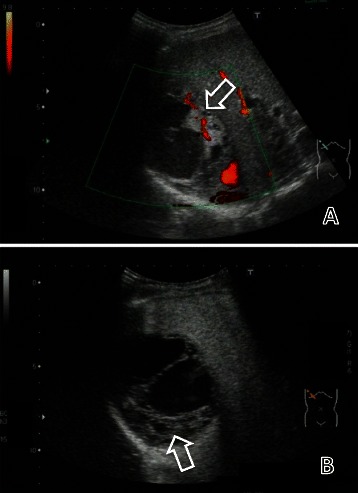
**Color Doppler and contrast-enhanced ultrasonography showing the multilocular cystic liver mass.** Color Doppler ultrasonography **(A)** and vascular phase contrast-enhanced ultrasonography using Sonazoid (92 seconds after bolus injection) **(B)** showed the multilocular cystic mass consisted of thickened irregular septa and nodal walls with blood flow.

**Figure 3:**
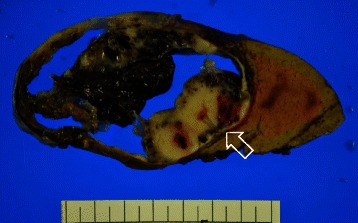
**Gross findings of the resected multilocular cystic liver tumor.** The multilocular cystic liver tumor measured 8 × 7 × 6 cm in size, and consisted of thickened irregular septa and white mural nodules (arrow).

**Figure 4:**
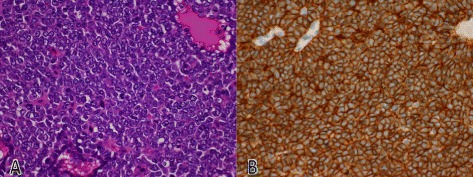
**Microscopic findings of the multilocular cystic liver tumor.** Histopathological examination revealed monotonous solid and sheet-like proliferation of small- to medium-sized round cells with rosette formation (**A**, H&E, ×100). Immunohistochemical staining for CD99 was positive (**B**, ×100).

The patient was transferred to our hospital three weeks later. Color Doppler ultrasonography showed the multilocular cystic mass consisted of thickened irregular septa and nodal walls with blood flow (Figure 2A). Contrast-enhanced ultrasonography using Sonazoid (Daiichi-Sankyo, Tokyo, Japan) described the enhancement of septa and nodal walls in the vascular phase to the same degree as the surrounding parenchyma (Figure 2B). Magnetic resonance imaging was performed but provided no additional information. We made a presumed diagnosis of mucinous cystadenoma of the liver based on the imaging studies and the patient underwent an extended right hepatectomy in August 2012 (Figure 3) with no postoperative complications including hepatic failure. R0 resection was achieved. Histopathological examination revealed monotonous solid and sheet-like proliferation of small- to medium-sized round cells with rosette formation (Figure 4A). Tumor cells were positive for periodic acid-Schiff (PAS) reaction and immunoreactive for CD99 (Figure 4B), NKX2.2 and the neuroendocrine markers CD56 and synaptophysin. EWS-FLI-1 fusion transcript type 1, which is the result of translocation between exon 7 of the EWSR1 gene on chromosome 22q12 and exon 6 of the FLI1 gene on chromosome 11q24, was detected by reverse transcriptase real-time polymerase chain reaction. Pathological and molecular analysis confirmed the diagnosis of Ewing sarcoma. Postoperative technetium bone scintigraphy and fluorodeoxyglucose positron emission tomography revealed no bone or soft tissue lesion as a primary site of Ewing sarcoma, no residual lesion in the liver, and no distant metastases. Also, bone marrow aspiration and biopsy revealed no malignant cells. These findings supported a diagnosis of localized Ewing sarcoma arising in the liver. The patient received adjuvant systemic chemotherapy as follows: VDC (vincristine 1.4 mg/m2, doxorubicin 75 mg/m2, and cyclophosphamide 1200 mg/m2 followed by mesna) alternating with IE (ifosfamide 1.8 g/m2 with mesna each day for five days, and etoposide 100 mg/m2 for five days) until September 2013. We found no evidence of recurrence 15 months after completing chemotherapy.

## Discussion

Approximately 80% of EFTs in the pediatric population arise in bone and < 20% occur in soft tissues, while > 50% of adult EFTs occur in soft tissues, including the trunk (14%), retroperitoneum or intra-abdominal tissues (14%), viscera (8%), and other sites (21%) [10]. Visceral ES is reported to occur in the kidney [3], uterus [4], and gastrointestinal tract [5]. Patient characteristics of extraosseous ES are similar to those of other EFTs, and approximately 80% of patients are younger than 20 years [11].

To our knowledge, only three published case reports discuss ES arising in the liver [6–8] and because of the rarity of this entity, little information on the imaging features is available. Two previous case reports described very large solid tumors without cystic lesions on CT [6,7], and a third case report described an enlarged liver without mass lesion on CT scan [8]; CT scan in our case showed a multilocular cystic lesion. These findings indicate that CT scans in patients with ES arising in the liver can reveal a polycystic tumor, solid tumor, or an enlarged liver without mass lesion.

Cystic liver lesions generally represent a heterogeneous group of diseases. Simple solitary cyst, polycystic disease, parasitic cyst, neoplastic (primary or metastatic) disease, duct related disease, false cyst, and ciliated foregut cysts are classified as hepatic cysts [12]. Although most liver cysts are found incidentally and tend to be benign, some are associated with malignant disease and thick or irregular cyst walls and/or multilocular cyst space on imaging studies are suspicious for malignancy. In our case, the multilocular cystic liver mass consisted of thickened, irregular septa and nodal walls and these findings were highly suspicious for a malignant neoplasm. The preoperative clinical diagnosis in our case was mucinous cystadenoma, which is a rare cystic tumor that occurs within the liver parenchyma or in the extrahepatic bile ducts. Differential diagnoses for neoplastic liver cysts are mucinous cystadenoma, mucinous cystadenocarcinoma, hepatocellular carcinoma, and metastasis from other malignant disease [13]. ES is an uncommon differential diagnosis for cystic liver disease.

ES is described histologically as small, blue, round cells with hyperchromatic nuclei and scant cytoplasm. The morphological appearance of ES is similar to that of other small, blue, round cell tumors, including lymphoma, mesenchymal chondrosarcoma, medulloblastoma, desmoplastic small round cell tumors, and rhabdomyosarcoma. PAS stain is usually positive for ES because of the presence of abundant glycogen [14] and the vast majority of ES express strong cell surface glycoprotein CD99. Our case was also positive for PAS, CD99, vimentin, and NKX2.2 [15]. However, immunohistochemical findings are not sufficient for diagnosis because these findings are not specific for ES. Chromosomal translocation such as t(11;22)(q24;q12) is positive in > 85% of ES cases and also for PNET [16]. Approximately 10% of ES cases have other analogous translocations such as t(21;22)(q22;q12) and t(7;22)(q22;q12) [17,18]; therefore, cytogenetic and molecular techniques are useful to distinguish ES/PNET from other tumors.

The standard treatment for extraosseous ES is not established because of its rarity; therefore, we used the same strategy as for osseous ES [19]. Modern treatment guidelines recommend primary treatment with multi-agent chemotherapy followed by local treatment and additional chemotherapy [20]. ES is highly sensitive to chemotherapy with vincristine, doxorubicin, cyclophosphamide, ifosfamide and etoposide (VDC/IE). In our case, preoperative biopsy was not diagnostic and surgical resection was performed as an initial treatment with six cycles of VDC/IE chemotherapy planned after complete resection. Current guidelines recommend physical examination, imaging of the primary site and chest, complete blood count, and other laboratory studies every 2–3 months for 24 months, with increasing testing intervals after 24 months and annually after five years [20].

## Conclusions

To our knowledge, this is the fourth reported case of Ewing sarcoma occurring in the liver, and the first case with a multilocular cystic liver mass. Imaging examinations of the other three reported cases showed solid tumors and a diffusely enlarged liver without a mass lesion. Clinicians should consider the possibility of ES in young patients with a multilocular cystic liver mass with thick and/or irregular cyst walls.

## Consent

Written informed consent was obtained from the patient for publication of this case report and any accompanying images. A copy of the written consent is available for review by the Editor of this journal.

## Abbreviations

EFTs: Ewing sarcoma family of tumors
ES: Ewing sarcoma
PNET: Primitive neuroectodermal tumor
CT: Computed tomography
VDC: Vincristine, doxorubicin and cyclophosphamide
IE: Ifosfamide and etoposide
